# Economic evaluations of angiotensin-converting enzyme inhibitors and angiotensin II receptor blockers in type 2 diabetic nephropathy: a systematic review

**DOI:** 10.1186/1471-2369-15-15

**Published:** 2014-01-15

**Authors:** Yunyu Huang, Qiyun Zhou, Flora M Haaijer-Ruskamp, Maarten J Postma

**Affiliations:** 1 Department of Pharmacy, Unit of Pharmaco Epidemiology & Pharmaco Economics, University of Groningen, Groningen, The Netherlands; 2 Department of Clinical Pharmacology, University Medical Center Groningen, University of Groningen, Groningen, The Netherlands; 3 School of Public Health, Fudan University, Shanghai, China

## Abstract

**Background:**

Structured comparison of pharmacoeconomic analyses for ACEIs and ARBs in patients with type 2 diabetic nephropathy is still lacking. This review aims to systematically review the cost-effectiveness of both ACEIs and ARBs in type 2 diabetic patients with nephropathy.

**Methods:**

A systematic literature search was performed in MEDLINE and EMBASE for the period from November 1, 1999 to Oct 31, 2011. Two reviewers independently assessed the quality of the articles included and extracted data. All cost-effectiveness results were converted to 2011 Euros.

**Results:**

Up to October 2011, 434 articles were identified. After full-text checking and quality assessment, 30 articles were finally included in this review involving 39 study settings. All 6 ACEIs studies were literature-based evaluations which synthesized data from different sources. Other 33 studies were directed at ARBs and were designed based on specific trials. The Markov model was the most common decision analytic method used in the evaluations. From the cost-effectiveness results, 37 out of 39 studies indicated either ACEIs or ARBs were cost-saving comparing with placebo/conventional treatment, such as amlodipine. A lack of evidence was assessed for valid direct comparison of cost-effectiveness between ACEIs and ARBs.

**Conclusion:**

There is a lack of direct comparisons of ACEIs and ARBs in existing economic evaluations. Considering the current evidence, both ACEIs and ARBs are likely cost-saving comparing with conventional therapy, excluding such RAAS inhibitors.

## Background

Approximately one fourth to one third of patients with diabetes mellitus develop renal manifestations [1-4]. Clinical stages of diabetic nephropathy are generally categorized into stages based on the values of urinary albumin excretion: microalbuminuria (MiA) and macroalbuminuria (MaA) [5]. The prevalence of MiA and MaA in type 2 diabetes is as high as 37–40% in western countries and 57.4–59.8% in Asian countries [6-8]. 20–40% of type 2 diabetic patients with MiA progress to overt nephropathy, and by 20 years after onset of overt nephropathy, about 20% will have progressed to end-stage renal diseases (ESRD) [9]. Because of the large prevalence, diabetes has become the most common single cause of ESRD in the U.S. and Europe [10,11]. As therapies and interventions for coronary artery disease continue to improve, more patients with type 2 diabetes may be expected to survive long enough to develop renal failure.

In developed countries, ESRD is a major cost driver for health-care systems, with annual growth of dialysis programs ranging between 6% and 12% over the past two decades and continuing to grow, particularly in developing countries [12]. Although there are no definitive cure solutions, there is good evidence that adequate treatment can delay or prevent the progress of diabetic nephropathy including strict control of glycaemia, early treatment of hypertension, dietary protein restriction and lipid-lowering therapy [13]. Targeting renin–angiotensin–aldosterone system (RAAS) is the most effective way to delay renal disease progression. Treatment guidelines therefore recommended angiotensin-converting enzyme inhibitors (ACEIs) and angiotensin II receptor blockers (ARBs) as the first-choice agents for treating nephropathy in diabetic patients [14].

Both ACEIs and ARBs target the RAAS and have proven their renal protective effects in diabetic patients in various clinical trials. One disadvantage of ACEIs [15-17] in comparison with ARBs is the higher risk of dry cough while significant differences in effectiveness between these two drug classes have not been shown convincingly although ARBs have been more thoroughly investigated in controlled settings in the recent decade providing relatively high levels of evidence. Often clinical practice guidelines recommend both ACEIs and ARBs in diabetic patients with or even without (micro)albuminuria [18].

Pharmacoeconomic evaluations of ACEIs and ARBs have been widely applied based on clinical trials’ results. The pharmacoeconomic results of ARBs have been reviewed previously [19-26]. ARBs were suggested to be cost saving in type 2 diabetic patients with nephropathy versus conventional therapy, largely due to the high costs of treatment of ESRD. However, a systematic review of cost-effectiveness results of ACEIs in type 2 diabetic patients with renal disease is still lacking. In addition, the need of a structured pharmacoeconomic comparison of the ACEIs with ARBs is pointed out by some researchers [21,26].

The aim of this study is to address the similarities and differences in cost-effectiveness analyses for both ACEIs and ARBs in type 2 diabetic patients with nephropathy. In particular, three objectives are addressed: 1) to summarize the cost-effectiveness of ACEIs; 2) to update the cost-effectiveness of ARBs; 3) to compare the characteristics of different economic evaluations and analyze potential differences and similarities in the cost-effectiveness between the two drug classes reviewed.

## Methods

### Literature search strategy

A systematic literature search was performed in MEDLINE and EMBASE for the period November 1, 1999 to Oct 31, 2011. The key words (MeSH headings in MEDLINE, EMtree terms in EMBASE and other text terms) included were (Table 1):

– Indicating target drugs, the variations in and abbreviations of ACEIs and ARBs were searched, such as ‘angiotensin receptor antagonists’ , ‘renin angiotensin aldosterone system inhibitors’, and specific drug names of different ACEIs or ARBs, including 10 specific ACEIs (such as captopril, enalapril, etc.) and 8 ARBs (such as losartan, irbesartan, etc.).

– Indicating diabetic nephropathy, key words were limited to ‘type 2 diabetes’ and its variations. Variations of nephropathy were combined with diabetes, such as ‘diabetic renal diseases’ or ‘diabetic kidney diseases’.

– Indicating economic evaluations, various key words relating to different evaluation types, pharmacoeconomics, cost of drugs and cost analysis were searched, including ‘cost-effectiveness analysis’ (CEA), ‘cost-utility analysis’ (CUA), ‘cost-benefit analysis’ (CBA), and ‘cost savings’, etc.

**Table 1:** Search terms for systematic review

**Search terms**	**MEDLINE**	**EMBASE**
**Drug**	**Mesh:** Angiotensin-Converting Enzyme Inhibitors; Angiotensin Receptor Antagonists;	**EMtree:** dipeptidyl carboxypeptidase inhibito; angiotensin receptor antagonist;
	**TIAB (Title and Abstract):** ACEIs; ARBs; ACEI; ARB; renin angiotensin system inhibitor*^a^; renin angiotensin aldosterone system inhibitor*; ACE inhibitor*; RAS inhibitor*; RAAS inhibitor*; angiotensin converting enzyme inhibitor*; renin angiotensin system inhibitor*; angiotensin receptor blocker*; Losartan; Candesartan; Valsartan; Irbesartan; Telmisartan; Eprosartan; Olmesartan; Azilsartan; Benazepril; Captopril; Enalapril; Fosinopril; Lisinopril; Moexipril; Perindopril; Quinapril; Ramipril; Trandolapril	**ab,ti (Abstract and Title):** angiotensin receptor blocker; angiotensin receptor blockers; arb; arbs; ace inhibitor; ace inhibitors; angiotensin converting enzyme inhibitor; angiotensin converting enzyme inhibitors; angiotensin converting enzyme (ace) inhibitor; angiotensin converting enzyme (ace) inhibitors; acei; aceis; renin angiotensin system inhibitor; renin angiotensin system inhibitors; renin angiotensin system (ras) inhibitor; renin angiotensin system (ras) inhibitors; ras inhibitor; ras inhibitors; renin angiotensin aldosterone system inhibitor; renin angiotensin aldosterone system inhibitors; raas inhibitor; raas inhibitors; losartan; candesartan; valsartan; irbesartan; telmisartan; eprosartan; olmesartan; azilsartan; benazepril; captopril; enalapril; fosinopril; lisinopril; moexipril; perindopril; quinapril; ramipril; trandolapril;
**Diabetic Nephropathy (DN)**	**Mesh:** Diabetes Mellitus, Type 2; Diabetic Nephropathies; Kidney Failure, Chronic;	**EMtree:** non insulin dependent diabetes mellitus; diabetic nephropathy;
	**TIAB:** diabetic nephropathy*; diabetic renal disease*; diabetic kidney disease*;	**ab,ti:** diabetic nephropathy; diabetic nephropathies; diabetic renal diseases; diabetic renal disease; diabetic kidney diseases; diabetic kidney disease
**Economic Evaluation (EE)**	**Mesh:** Economics, Pharmaceutical; Costs and Cost Analysis; Drug Costs; Cost Savings; Cost of Illness; Cost-Benefit Analysis;	**EMtree:** pharmacoeconomics; economic evaluation; drug cost; cost control; cost of illness; cost benefit analysis; cost effectiveness analysis;
	**TIAB:** cost effect*; cost utility; cost benefit*; economic evaluation*; cost analys*	**ab,ti:** cost effectiveness; cost utility; cost benefit; economic evaluation; economic evaluations; cost analys;
**Search Strategy**	(“Drug Term 1”[Mesh] **OR** “Drug Term 2”[TIAB] …) **AND** (“DN Term 1”[Mesh] **OR** “DN Term 2”[TIAB] …) **AND** (“EE Term 1”[Mesh] OR “EE Term 2”[TIAB] …)	(‘Drug Term 1’/exp **OR** ‘Drug Term 2’:ab,ti …) **AND** (‘DN Term 1’/exp **OR** ‘DN Term 2’:ab,ti …) **AND** (‘EE Term 1’/exp **OR** ‘EE Term 2’:ab,ti …) **NOT [medline]/lim**^**b**^

a: An asterisk (*) following the word is the wildcard character, which means to search in MEDLINE for all terms that begin with a word; b: To exclude articles that can be found in MEDLINE.

The references of identified articles were manually screened for relevant economic evaluations not identified in the above-mentioned searches (snowballing).

### Study selection

Inclusion criteria for the review were as follows (following the PICOS-design):

– **P**opulation: patients in studies had to have type 2 diabetes with symptoms of renal diseases;

– **I**nterventions and **C**omparators: studies must examine an ACEI- or ARB-based treatment regimen for the progression of diabetic nephropathy compared with regimens that did not include these medications, or if available, compare ACEIs with ARBs directly;

– **O**utcomes: clinical outcomes should be relevant to renal disease symptoms, including overt diabetic nephropathy, ESRD (kidney transplantation or dialysis), all-cause mortality, etc.; and

– **S**tudy design: studies had to be original economic evaluations.

Other criteria concerned that studies had to have been published as full-length articles and were peer-reviewed for English-language journals.

Study selection was performed in three rounds. First, titles and abstracts of searched articles were scanned and checked. In the second round, the full-texts of included articles were read carefully and quality was assessed in the last round. Two authors independently assessed the quality of the articles included and extracted the data. Differences were resolved by consensus.

### Quality assessment

Quality assessment was conducted at the ‘study’ level, i.e. each study was analyzed one by one. A checklist for critical appraisal of economic evaluations [27] was used to evaluate the study quality. The checklist comprises 12 criteria assessing the study design, outcomes and costs and the extrapolation of the results of an economic evaluation. An additional file shows this checklist in more detail (see Additional file 1).

The criterion ‘applicable to local population’ was not included in the assessment as we didn’t felt this was relevant for the current study; i.e. 11 criteria were considered in the end. In case studies showing cost savings, the absence of an explicit incremental cost-effectiveness ratio (ICER) was classified as adequate, since in that case no incremental ratio is necessary or meaningful.

Studies were subsequently included in the full review if: 1) the outcomes and costs have been assessed as being credibly, 2) at least 6 of the 11 quality criteria were rated as adequate or good; and 3) not more than three quality criteria were assessed as being inadequate.

### Data extraction

Data extraction was based on the 11 criteria included in the quality assessment checklist which concerned: 1) basic information of study design; 2) data on outcomes and costs; and 3) results and conclusions. We grouped articles into two groups, reflecting ACEIs and ARBs. The latter group was subdivided into three subgroups in line with the three mostly analyzed ARBs, irbesartan, losartan and valsartan.

To make the results comparable across the studies, cost-saving or ICER results were standardized to 2011 price levels, by applying the appropriate annual deflators for each country, based on the statistics from the World Bank [28]. Since the deflator data for Taiwan was not available from the World Bank, cost data of this region was not standardized. The original cost-saving result was showed as reference.

All the currencies were converted to 2011 Euros, based on the Euro rate as of June 30th, 2011 [29].

The results of selected studies were classified in 5 categories: 1) cost-saving: net life years or QALYs gained in conjunction with ≥ €1,000 saved per patient as compared with the comparison intervention; 2) almost cost-neutral: net life years or QALYs gained, with < €1,000 saved per patient; 3) very cost-effective: 0 < ICER ≤ €20,000; 4) cost-effective: €20,000 < ICERs ≤ €40,000; 5) not cost-effective: ICERs > €40,000. The classification was based on both literature and suggestions in identified studies in this review [30,31].

## Results

**Figure 1:**
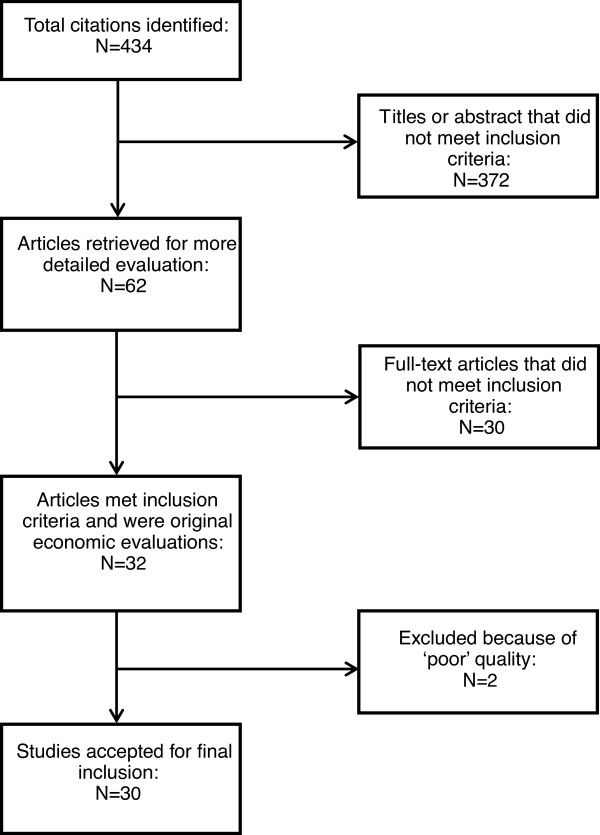
Flow chart summarizing systematic study selection process.

Up to October 2011, 434 articles (141 articles from PubMed and 293 articles from EMBASE) were identified. After full-text checking, 32 articles were included into the quality assessment. After quality assessment, 30 articles were finally included in this review (Figure 1). One of the excluded articles had 4 criteria assessed as inadequate and only 4 criteria assessed as good. The other one merely got 5 criteria rated as adequate among the 11 criteria considered.

Among these 30 selected articles, in one article on losartan for an Asian population [32] only the data from Hong Kong were considered as the cost data from other Asian countries or regions assessed seemed not to be of adequate quality. Finally, 39 studies in different countries or regions contained in these 30 articles were included in the analysis.

### Summary of selected studies

Table 2 summarizes the basic features of studies included. All six ACEIs studies [33-38] were literature-based evaluations which synthesized data from different sources. All ARBs studies [32,39-62] were designed based on specific trials. The Markov model was the most common decision analytic method used in these evaluations. From the cost-effectiveness results, 37 out of 39 studies indicated both ACEIs and ARBs were cost-saving comparing with placebo/conventional treatment or amlodipine. In the absence of clear cost savings, cost neutrality of very favorable cost-effectiveness was achieved minimally. No studies were identified with a direct cost-effectiveness comparison between ACEIs and ARBs.

**Table 2:** Summary of selected studies (number of study)

		**ACEIs (total 6)**	**ARBs (total 33)**	**ARBs Losartan (total 14)**	**ARBs Irbesartan (total 18)**	**ARBs Valsartan (total 1)**
Data source	Trial based	0	33	14	18	1
	Literature based	6	0	0	0	0
Intervention and control group	Comparing with placebo/conventional therapy	2	22	14	8	0
	Comparing with other drugs	0	12	0	11	1
	Comparing different strategies	4	10	0	10	0
Decision model	Markov model	6	20	1	18	1
	Weibull model	0	3	3	0	0
	Regression method	0	10	10	0	0
Perspective	Third party payer	4	33	14	18	1
	Societal	2	0	0	0	0
CE results	Cost-saving	5	32	13	18	1
	Cost-neutral	0	1	1	0	0
	Very cost-effective	1	0	0	0	0
	Cost-effective	0	0	0	0	0
	Not cost-effective	0	0	0	0	0

### Design of selected studies

Key features of the design of the selected studies were summarized in Table 3. Six studies of ACEIs [33-38] were diverse in data sources, intervention and control groups. The 33 studies on ARBs showed much more consistency within each ARB drug class (losartan, irbesartan and valsartan) regarding control and data sources concerning the various clinical trials done in ARBs.

**Table 3:** Study design of economic evaluations on ACEIs and ARBs

**Study, country/region**	**Source of effectiveness data**	**Intervention group**	**Control group**	**Decision model type**	**Time horizon (years)**	**Evaluation type**
**ACEIs**						
Golan et al. 1999 US [33]	UERNN, LEAPP and EADN trial	‘Treat all’ strategy^a^	(1) Screen for MiA^b^;	Markov model with 5 states	10	CEA & CUA (Life-years & QALYs)
			(2) Screen for gross proteinuria^c^.			
Sakthong et al. 2001 Thailand [34]	LEAN trial and the opinion of nephrologists	Enalapril at the dose of 10 mg/day	Placebo	Markov model with 4 stages	25	CEA (Life years)
Rosen et al. 2005 US [35]	UERNN, EADN, LEAN, H-MH studies and HOPE trial	Medicare first-dollar coverage of ACEIs	Year 2005’s Medicare practice	Markov model adding a cardiovascular events component.	lifetime	CEA & CUA (Life-years & QALYs)
Campbell et al. 2007 US [36]	UERNN, EADN, H-MH studies and IRMA-2 trial	ACEI therapy in normoalbuminimuric, microalbuminuric, and macroalbuminuric patients	No ACEI initiation in patients	Markov model	8	CEA (CVD event avoided, life saved, dialysis prevented, composite endpoint avoided)
Adarkwah et al. 2010 Germany [37]	EADN and two meta-analyses	‘Treat all’ strategy^a^	(1) Screen for MiA^b^;	Markov model with 5 states	50	CUA (QALY)
			(2) Screen for MaA^c^;			
			(3) no-screening and no-treatment alternative.			
Adarkwah et al. 2011 Netherlands [38]	EADN and two meta-analyses	‘Treat all’ strategy^a^	(1) Screen for MiA^b^;	Markov model with 5 states	50	CUA (QALY)
			(2) Screen for MaA^c^.			
**ARBs**						
**Losartan**						
Herman et al. 2003 US [39]	RENAAL trial	Losartan	Placebo^d^	A regression-based method	3.5 / 4	CEA (Number of ESRD days)
Souchet et al. 2003 France [40]	RENAAL trial	Losartan (initial daily dosing of losartan was 50 mg, with the possibility of titration to 100 mg/day)	Placebo^d^	A regression-based method	3.5 / 4	CEA (Number of ESRD days)
Burgess et al. 2004 Canada [41]	RENAAL trial	Losartan	Placebo^d^	A regression-based method	3.5 / 4	CEA (Number of ESRD days)
Szucs et al. 2004 Switzerland [42]	RENAAL trial	Losartan (initial daily dosing of losartan was 50 mg, with the possibility of titration to 100 mg/day)	Placebo^d^	A regression-based method	3.5 / 4	CEA (Number of ESRD days)
Seng et al. 2005 Hong Kong [32] (only data of Hong Kong were included)	RENAAL trial	Losartan	Placebo^d^	A regression-based method	3.5	CEA (Number of ESRD days)
Arredondo et al. 2005 Mexico [43]**]**	RENAAL trial	Losartan	Placebo^d^	A variation of the cumulative incidence competing risk method / Weibull model	25 (life time)	CEA (Cumulative incidence of ESRD, life expectancy)
Vora et al. 2005 UK [44]	RENAAL trial	Losartan (50–100 mg QD)	Conventional antihypertensive treatment^d^ (excluding ACEIs or angiotensin II antagonists)	Weibull model	life time	CEA (Cumulative incidence of ESRD, life expectancy)
Carides et al. 2006 US [45]	RENAAL trial	Losartan	Placebo^d^	A cumulative incidence competing risk method / Weibull model	25 (life time)	CEA (Cumulative incidence of ESRD, life expectancy)
Stafylas et al. 2007 Greece [46]	RENAAL trial	Losartan (50–100 mg QD)	Placebo^d^	Markov model with 6 states	3.5/4	CEA (Number of ESRD days)
de Portu et al. 2011 Italy, France, Germany, Switzerland, US [47]	RENAAL trial	Losartan	Standard care^d^	Standard methods by comparing the economic outcomes deriving from additional losartan to standard care vs standard care alone	3.4	CEA (Number of ESRD days)
**Irbesartan**						
Rodby RA et al. 2003 US [48]	IDNT trial	Irbesartan titrated from 75 to 300 mg/day	(1) ‘Control’^d^;	Markov model with 5 stages	25	CEA (Life expectancy)
			(2) Amlodipine titrated from 2.5 to 10 mg/day.			
Palmer AJ et al. 2003 Belgium, France [49]	IDNT trial	Irbesartan titrated from 75 to 300 mg/day	(1) ‘Control’^d^;	Markov model with 5 stages	25	CEA (Life expectancy)
			(2) Amlodipine titrated from 2.5 to 10 mg/day.			
Coyle D et al. 2004 Canada [50]	IDNT trial	Irbessartan	(1) Amlodipine;	Markov model with 5 stages	25	CEA (Life expectancy)
			(2) Standard care^d^			
Palmer AJ et al. 2004 UK [51]	IDNT trial	Irbesartan 300 mg per day	(1) ‘Control’^d^;	Markov model with 5 stages	25	CEA (Life expectancy)
			(2) Amlodipine 10 mg per day.			
Palmer AJ et al. 2004 US [52]	IRMA-2 study and IDNT	‘Early irbesartan’^e^	(1) ‘Control’^d^;	Markov model with 7 stages	25	CEA (Years free of ESRD, cumulative incidence ESRD, life expectancy)
			(2) ‘Late irbesartan’^f^			
Palmer AJ et al. 2005 Spain [53]	IRMA-2 study and IDNT	‘Early irbesartan’^e^	Standard antihypertensive medications^d^	Markov model with 7 stages	25	CEA (Years free of ESRD, cumulative incidence ESRD, life expectancy)
Palmer AJ et al. 2006 Switzerland [54]	IRMA-2 study and IDNT	‘Early irbesartan’^e^	Conventional antihypertensive treatment^d^ initiated when patients had developed MiA.	Markov model with 7 stages	25	CEA (Years free of ESRD, cumulative incidence of ESRD, life expectancy)
Palmer AJ et al. 2006 France [55]	IRMA-2 study and IDNT	‘Early irbesartan’^e^	(1) ‘Control’^d^;	Markov model with 7 stages	25	CEA & CUA (Years free of ESRD, life expectancy, QALY)
			(2) ‘Late irbesartan’^f^			
Palmer AJ et al. 2007 Hungary [56]	IRMA-2 study and IDNT	‘Early irbesartan’^e^	‘Placebo’^d^: standard antihypertensive medications initiated when patients developed MiA.	Markov model with 7 stages	25	CEA (Years free of ESRD, cumulative incidence ESRD, life expectancy)
Palmer AJ et al. 2007 UK [57]	IRMA-2 study and IDNT trial	‘Early irbesartan’^e^	(1) ‘Control’^d^;	Markov model with 7 stages	25	CEA (Years free of ESRD, cumulative Incidence of ESRD, life expectancy)
			(2) ‘Late irbesartan’^f^			
Coyle D et al. 2007 Canada [58]	IRMA-2 study and IDNT	‘Early irbesartan’^e^	(1) ‘Late irbesartan’^f^;	Markov model with 7 stages	25	CEA (Life expectancy)
			(2) ‘Conventional’^d^			
Yang W.C. et al. 2007 Taiwan [59]	IRMA-2 study and IDNT	‘Early irbesartan’^e^	(1) ‘Standard’^d^;	Markov model with 7 stages	25	CEA (Life expectancy, number of years free of ESRD, cumulative incidence of ESRD)
			(2) ‘Late irbesartan’^f^;			
			(3) ‘Late amlodipine’^g^			
Annemans et al. 2008 China, Taiwan, Malaysia, Thailand, South Korea [60]	IRMA-2 study and IDNT trial	‘Early irbesartan’^e^	(1) ‘Standard’^d^;	Markov model with 7 stages	25	CEA (Cumulative incidence of ESRD, number of days in dialysis, number of years free of ESRD, life expectancy)
			(2) ‘Late irbesartan’^f^;			
			(3) ‘Late amlodipine’^g^			
**Valsartan**						
Smith DG et al. 2004 US [61]	MARVAL study	Valsartan	Amlodipine	Markov model with 7 stages	8	CUA (Quality-adjusted survival)

a: no screening was performed at all and patients started on ACEI therapy at the time of diagnosing type 2 diabetes.

b: patients were screened for MiA once a year and ACEI treatment was started if the test result is positive.

c: patients were screened for MaA once a year and ACEI treatment was started if the test result is positive.

d: standard antihypertensive therapy alone, excluding the use of ACEIs, ARBs.

e: standard antihypertensive therapy plus administration of irbesartan 300 mg/d at the onset of MiA.

f: standard antihypertensive therapy plus administration of irbesartan 300 mg/d once the patients reach the advanced diabetic nephropathy stage.

g: standard antihypertensive therapy plus administration of amlodipine titrated from 5 to 10 mg/d once the patients reach the advanced diabetic nephropathy stage.

**UERNN** = Use of enalapril to attenuate decline in renal function in normotensive, normoalbuminuric patients with type 2 diabetes mellitus; **LEAPP** = Long-term stabilizing effect of angiotensin-converting enzyme inhibition on plasma creatinine and on proteinuria in normotensive type II diabetic patients; **EADN** = The effect of angiotensin-converting-enzyme inhibition on diabetic nephropathy; **LEAN** = Long-term renoprotective effect of angiotensin-converting enzyme inhibition in non-insulin-dependent diabetes mellitus; **H-MH** = Effects of ramipril on cardiovascular and microvascular outcomes in people with diabetes mellitus: results of the HOPE study and MICRO-HOPE substudy; **HOPE** = The Heart Outcomes Prevention Evaluation; **RENAAL** = The reduction of endpoints in non-insulin dependent diabetes mellitus with the angiotensin II antagonist losartan; **IDNT** = The irbesartan in diabetic nephropathy trial; **IMRA-2** = The irbesartan in reduction of microalbuminuria-2; **MARVAL** = The microalbuminuria reduction with valsartan.

**CEA** = cost-effectiveness analysis; **CUA** = cost-utility analysis.

#### ACEIs

Six studies [33-38] evaluated the cost-effectiveness of ACEIs, all using a Markov model as the method for decision modeling. The transition probabilities in these Markov models, i.e. the sources and sizes of effectiveness data in these studies, were diverse. All six studies obtained their effectiveness data from more than one RCT [63-67] or from meta-analyses [37,38]. Only one of the studies [34] included a specific ACEI, enalapril, to compare with placebo, while the other five studies treated ACEIs as a group or drug class. ARBs were also included in the analytic model as a substitute for ACEIs when patients got cough side-effect in the two articles written by Adarkwah et al. [37,38].

#### ARBs

The 33 studies (included in 24 articles [32,39-61]) targeting ARBs have major similarities in study design. Fourteen evaluations for losartan [32,39-47] were based on The Reduction of Endpoints in Non-insulin Dependent Diabetes Mellitus with the Angiotensin II Antagonist Losartan (RENAAL) trial [62]. Eighteen evaluations of irbesartan [48-60] used data from the Irbesartan in Diabetic Nephropathy Trial (IDNT) [68] to assess the cost-effectiveness for patients with type 2 diabetes and overt nephropathy before 2004. Later the Irbesartan in Reduction of Microalbuminuria-2 (IRMA-2) [69] trial was added into the model to expand the progress of diabetic renal development from nephropathy back to the onset of MiA. The only study for valsartan was based on the MicroAlbuminuria Reduction With VALsartan (MARVAL) study [70].

All 14 losartan studies can be subdivided into two groups based on different time horizon. Eleven studies [32,39-42,46,47] were within-trial analyses, while the other three [43-45] extrapolated to beyond-trial time-horizon analyses. Ten within-trial analyses [32,39-42,47] used a straightforward method to calculate the effectiveness and cost. In this method, the patient-days spent in the stage of ESRD were estimated by subtracting the area under curve (AUC) of the Kaplan-Meier survival curve for time to the minimum of ESRD or all-cause death for both groups in the trial. The costs of ESRD were calculated by multiplying ESRD days and daily cost of ESRD. Only one within-trial study [46] performed a Markov model as the analytic method to evaluate the cost-effectiveness. Three beyond-trial studies [43-45] used a Weibull model to prolong the time horizon to lifetime. Cumulative incidence of ESRD and life expectancy were assessed as the effectiveness measurements.

Irbesartan for overt nephropathy was compared with conventional treatment and amlodipine in five studies [48-51]. These five studies were based on the IDNT trial and a Markov model with five stages (from ‘overt nephropathy’ via ‘double of serum creatinine’ , ‘ESRD + dialysis’ and ‘ESRD + transplant’ to ‘death’) was developed to evaluate life expectancy and lifetime cost. In particular, Palmer et al. combined the IRMA-2 trial with the IDNT trial and applied a seven-stage Markov model, extrapolating the Markov model with a previous MiA state [48,49,51-60]. ‘Early irbesartan’ (standard antihypertensive therapy plus irbesartan at the onset of MiA) was then compared with conventional therapy and ‘late irbesartan’ or ‘late amlodipine’ (standard antihypertensive therapy plus administration of irbesartan/amlodipine once the patients reach the advanced diabetic nephropathy stage).

Cost-effectiveness of Valsartan [61] was evaluated in one study using amlodipine as the control. A Markov model with seven stages was designed and QALYs were calculated as the effectiveness results.

### Cost-effectiveness results

The key features and main results of all included evaluations are summarized in Table 4.

**Table 4:** Main results of economic evaluations on ACEIs and ARBs

**Study, country/ region**	**Discount rate (per annum)**		**Perspective**	**Cost categories**	**Discounted life expectancy/QALY**	**Incremental cost per patients [year of value]**	**Incremental cost per patients (standardized to 2011 Euro)**	**CE with interventions**
	**Effects**	**Costs**						
	**(%)**	**(%)**						
**ACEIs**								
Golan et al. 1999 US [33]	3	3	Societal	The cost of ESRD (dialysis & transplant), ACEIs and screening	15.63 years/11.82 QALYs with ‘treat all’,	‘Treat all’ vs ‘screen for MiA’: $300^a^	‘Treat all’ vs ‘screen for MiA’: €299	Very cost-effective [‘Treat all’ *vs.* ‘screen for MiA’: €8,062/QALY]
					15.59 years/11.78 QALYs with ‘screen for MiA’, 15.39 years/11.59 QALYs with ‘screen for gross proteinuria’			
Sakthong et al. 2001 Thailand [34]	8	8	Not mentioned	The cost of ESRD (haemodialysis) and ACEI	9.04 years with enalapril, 7.54 years with control	-$1,198 [1999]	-€1,269	Cost saving [Enalapril]
Rosen et al. 2005 US [35]	3	3	Medicare and societal	(1) Medicare perspective: direct medical costs and future health care costs.	10.55 years/8.36 QALYs with Medicare first-dollar coverage of ACEIs, 10.30 years /8.13 QALYs with at the time practice	-$1,606 [2003]	-€1,453	Cost saving [Medicare first-dollar coverage of ACEIs]
				(2) Societal perspective: additional analyses included productivity gains and losses, caregiver time costs				
Campbell et al. 2007 US [36]	3	3	Health payer	Direct medical costs of nephropathy, CVD, and ACEIs		-$772 for normoalbuminuria on diagnosis, -$7,098 for MiA on diagnosis, $7,987 for MaA on diagnosis [2005]	-€658 for normoalbuminuria on diagnosis, -€6,048 for MiA on diagnosis, €6,806 for MaA on diagnosis	Cost-neutral [ACEIs used on normoalbuminuria]
								Cost saving [ACEIs on MiA]
Adarkwah et al. 2010 Germany [37]	3	3	the German statutory health insurance	The cost of ESRD (dialysis & transplant), ACEIs, ARBs and screening	15.21 QALYs with ‘treat all’,	‘Treat all’ vs. ‘placebo’: -€16,024 [2006]	‘Treat all’ *vs. ‘*placebo’: -€16,841	Cost saving [Treat all using ACEIs]
					15.14 QALYs with ‘screen for MiA’, 14.83 QALYs with ‘screen for MaA’, 14.46 QALYs with ‘placebo’			
Adarkwah et al. 2011 Netherlands [38]	1.5	4	Health care	The cost of ESRD (dialysis & transplant), ACEIs, ARBs and screening	19.63 QALYs with ‘treat all’, 19.54 QALYs with ‘screen for MiA’, 19.15 with ‘screen for MaA’	‘Treat all’ vs. ‘screen for MiA’: -€2,719, ‘treat all’ vs. ‘screen for MaA’: -€12,356 [2010]	‘Treat all’ *vs.* ‘screen for MiA’: -€2,749, ‘treat all’ *vs.* ‘screen for MaA’: -€12,492	Cost saving [Treat all using ACEIs]
**ARBs**								
**Losartan**								
Herman WH et al., 2003 US [39]	none	3	Health care system	The cost of ESRD (hemodialysis) and losartan therapy		Over 3.5 years: -$3,522 [2001]	Over 3.5 years: -€3,306	Cost saving [losartan]
Souchet T et al., 2003 France [40]	none	8.1%^b^	French health care system	The cost of ESRD (dialysis) and losartan therapy		Over 3.5 years: -€3,863 [2002]	Over 3.5 years: -€4,522	Cost saving [losartan]
Burgess ED et al., 2004 Canada [41]	none	none	Health care system	The cost of ESRD (dialysis & transplant) and losartan therapy		Over 3.5 years: -$3,675^a^	Over 3.5 years: -€3,368	Cost saving [losartan]
Szucs TD et al., 2004 Switzerland [42]	none	none	Swiss health care payer	The cost of ESRD (dialysis & transplant) and losartan therapy (only the insurance-paid part)		Over 3.5 years: -CHF4,084^a^	Over 3.5 years: -€3,660	Cost saving [losartan]
Seng WK et al., 2005 Hong Kong [32] (only data of Hong Kong were included)	3	3	Health care system	The cost of ESRD (dialysis) and losartan therapy		-$515 [2004]	-€413	Cost-neutral [losartan]
Arredondo A et al., 2005 Mexico [43]	3	3	Health care system	The cost of ESRD (dialysis), diabetes and losartan therapy	0.697 life years gained for losartan	-M$24,073 [2004]	-€1,861	Cost saving [losartan]
Vora J et al., 2005 UK [44]	3.5	3.5	The UK National Health Service (NHS)	The cost of ESRD (dialysis) and losartan therapy	7.82 life years with losartan, 7.38 life years with placebo (0.44 life years gained for losartan)	-£6,622 [2004]	-€9,182	Cost saving [losartan]
Carides GW et al., 2006 US [45]	3	3	Health care system	The cost of ESRD (dialysis), diabetes and losartan therapy	0.697 life years gained for losartan	-$24,632 [2002]	-€22,757	Cost saving [losartan]
Stafylas PC et al., 2007 Greece [46]	3	3	The Greek social insurance system	The cost of ESRD (dialysis & transplant) and 75% of drug treatment costs		Over 3.5 years: -€1,665.43 [2003]	Over 3.5 years: -€2,079	Cost saving [losartan]
de Portu S et al., 2011 Italy [47]	3	3	National Health care Service	The cost of ESRD (hemodialysis) and losartan therapy		-€3,602.98 [2009]	-€3,664	Cost saving [losartan]
de Portu S et al., 2011 France [47]	3	3	Health Insurance	The cost of ESRD (hemodialysis) and losartan therapy		-€4,531.35 [2009]	-€4,641	Cost saving [losartan]
de Portu S et al., 2011 Germany [47]	3	3	Health Insurance	The cost of ESRD (hemodialysis) and losartan therapy		-€3,019.66 [2009]	-€3,062	Cost saving [losartan]
de Portu S et al., 2011 Switzerland [47]	3	3	Medical Insurance	The cost of ESRD (hemodialysis) and losartan therapy		-€3,949.50 [2009]	-€3,977	Cost saving [losartan]
de Portu S et al., 2011 US [47]	3	3	Centers for Medicare & Medicaid Services	The cost of ESRD (hemodialysis) and losartan therapy		-€3,855.50 [2009]	-€4,007	Cost saving [losartan]
**Irbesartan**								
Rodby RA et al., 2003 US [48]	3	3	Health care system	The cost of ESRD (dialysis & transplant), hospitalizations, irbesartan & concomitant antihypertensive drugs	8.225 years with irbesartan, 7.484 years with control (0.741 years gained for irbesartan)	-$15,607 [2000]	-€14,987	Cost saving [irbesartan]
Palmer AJ et al., 2003 Belgium [49]	3	3	Institut National d’Assurance de Maladie et Invalidite’ (INAMI)	The cost of ESRD (dialysis & transplant) and irbesartan & concomitant antihypertensive drugs	8.57 years with irbesartan, 7.95 years with control (0.62 years gained for irbesartan)	-€11,885 [2002]	-€14,231	Cost saving [irbesartan]
Palmer AJ et al., 2003 France [49]	3	3	Social security	The cost of ESRD (dialysis & transplant) and irbesartan & concomitant antihypertensive drugs	8.58 years with irbesartan, 7.97 years with control (0.61 years gained for irbesartan)	-€16,345 [2002]	-€19,132	Cost saving [irbesartan]
Coyle D et al., 2004 Canada [50]	5	5	Third party payer	The cost of ESRD (dialysis & transplant), irbesartan & concomitant antihypertensive drugs and other medical costs	6.80 years with irbesartan, 6.37 years with control (0.43 years gained for irbesartan)	-CAD12,564 [2001]	-€11,457	Cost saving [irbesartan]
Palmer AJ et al., 2004 UK [51]	1.5	6	National Health Service (NHS) payer	The cost of ESRD (dialysis & transplant) and irbesartan & concomitant antihypertensive drugs	0.58 years gained for irbesartan vs control	-£4,978^a^	-€7,075	Cost saving [irbesartan]
Palmer AJ et al., 2004 US [52]	3	3	Third party reimbursement	The cost of ESRD (dialysis & transplant) and irbesartan	11.46 years with ‘early irbesartan’, 10.54 years with ‘late irbesartan’, 10.50 years with control (0.96 years gained for irbesartan vs control)	Early irbesartan vs. control: -$11,922, late irbesartan vs. control: -$3,252 [2000]	Early irbesartan vs. control: -€11,448, late irbesartan vs. control: -€3,123	Cost saving [early irbesartan]
Palmer AJ et al., 2005 Spain [53]	3	3	Third party payer	The cost of ESRD (dialysis & transplant) and irbesartan	12.37 years with ‘early irbesartan’, 11.53 years with control (0.84 years gained for irbesartan)	-€11,082^a^	-€12,971	Cost saving [early irbesartan]
Palmer AJ et al., 2006 Switzerland [54]	5	5	Third party Swiss health insurance payer	The cost of ESRD (dialysis & transplant) and irbesartan	10.37 years with ‘early irbesartan’, 9.80 years with control (0.57 years gained for irbesartan)	-CHF21,487 [2003]	-€19,257	Cost saving [early irbesartan]
Palmer AJ et al., 2006 France [55]	3	3	Third party French social security insurance payer	The cost of ESRD (dialysis & transplant) and irbesartan	12.17 years /10.55 QALYs with ‘early irbesartan’, 11.27 years /9.58 QALYs with ‘late irbesartan’, 11.23 years /9.52 QALYs with control (0.94 years /1.03 QALYs gained for irbesartan vs control)	‘Early irbesartan’ vs. control: -€22,314, ‘late irbesartan vs. control’: -€6,619 [2002]	‘Early irbesartan’ vs. control: -€26,119, ‘late irbesartan’ vs. control: -€7,748	Cost saving [early irbesartan]
Palmer AJ et al., 2007 Hungary [56]	5	5	Third-party Hungarian health insurance payer	The cost of ESRD (dialysis & transplant) and irbesartan	8.16 years with ‘early irbesartan’, 7.62 years with control (0.54 years gained for irbesartan)	-HUF519,993 [2002]	-€2,564	Cost saving [early irbesartan]
Palmer AJ, 2007 UK [57]	3.5	3.5	Third party UK National Health Service (NHS) payer	The cost of ESRD (dialysis & transplant) and irbesartan	11.00 years with ‘early irbesartan’, 10.20 years with ‘late irbesartan’, 10.18 years with control (0.82 years gained for irbesartan vs control)	‘Early irbesartan’ vs. control: -£3,801, ‘late irbesartan’ vs. control:- £1,491 [2002]	‘Early irbesartan’ vs. control: -€5,532, ‘late irbesartan’ vs. control: -€2,170	Cost saving [early irbesartan]
Coyle D et al., 2007 Canada [58]	5	5	Canadian health and social care system	All direct costs, including the costs of health, social services, long-term care.	11.52 years with ‘early irbesartan’, 11.06 years with ‘late irbesartan’, 10.90 years with control (0.62 years gained for irbesartan vs control)	‘Early irbesartan’ vs. control: -CAD68,400, ‘late irbesartan’ vs. control: -CAD14,300 [2006]	‘Early irbesartan’ vs. control: -€57,871, ‘late irbesartan’ vs. control: -€12,099	Cost saving [early irbesartan]
Yang W.C. et al., 2007 Taiwan [59]	3	3	Third-party payer in Taiwan (Taiwan National Health Insurance Program)	The cost of ESRD (dialysis & transplant) and irbesartan	12.003 years with ‘early irbesartan’, 11.332 years with ‘late irbesartan’, 11.223 years with control (0.780 years gained for irbesartan vs control)	‘Early irbesartan’ vs. control: -$7,603, ‘late irbesartan’ vs. control: -$3,233 [2004]		Cost saving [early irbesartan]
Annemans L et al., 2008 China, Taiwan, Malaysia, Thailand, South Korea [60]	5	5	Third party payer	The cost of ESRD (dialysis & transplant) and irbesartan	‘Early irbesartan’ strategy had the longest life expectancy (no detail data)	The least expensive strategy: ‘early irbesartan’ (no detail data)		Cost saving [early irbesartan]
**Valsartan**								
Smith DG et al., 2004 US [61]	3	3	Third-party payer	Medical care costs including costs of study drugs, routine health care services, and aggregate estimates of medical care associated with the various health states.	6.390 QALYs with valsartan, 5.835 QALYs with amlodipine (0.555 QALYs gained for valsartan)	-$32,412 [2001]	-€30,424	Cost saving [valsartan]

a: In which year the value of money standardized was not clear. It was assumed to be one year before the publication.

b: The total discount rate within time horizon, not annually.

#### ACEIs

Of the six ACEIs’ studies, two [33,35] adopted a societal perspective. This contained additional cost analyses including productivity gains and losses, caregiver time costs. The other four [34,36-38] took the third party payer/health care perspective including only direct costs of nephropathy, ACEIs or other related treatment such as those for cardiovascular disease (CVD). All studies except one [33] favored ACEIs due to the cost-saving results. The exception was the evaluation from Golan et al. [33], showing that compared to ‘screen for MiA’ (patients were screened for MiA once a year and ACEI treatment was started if the test result is positive), the ‘treat all’ strategy with ACEIs (no screening was performed at all and patients started on ACEI therapy at the time of diagnosing type 2 diabetes) raised the costs by $300, but the results still supported ‘treat all’ strategy as very cost-effective. –It should be noted that these positive results were based on the comparison between ACEIs and no blood pressure (BP) control treatment but not other BP control interventions.

#### ARBs

Based on the RENAAL trial, all the results over 3.5 years indicated losartan was cost-saving or cost-neutral (Hong Kong) [32] comparing to placebo/conventional therapy. The cost savings per patients ranged from €2,079 in Greece [46] to €4,641 in France [47]. When the time horizon was prolonged to lifetime or 25 years, beyond-trial studies showed that the net cost savings by adding losartan to conventional therapy were €9,182 in UK [44], €1,861 in Mexico [43] and €22,757 in U.S [45].

For irbesartan, results consistently showed cost-savings comparing with conventional therapy or amlodipine, even when already started at the onset of MiA. Such early start of irbesartan would economically be even more attractive as compared with late irbesartan starting at overt nephropathy. The five studies [48-51] based on the IDNT trial demonstrated that irbesartan for overt nephropathy could prolong life expectancy with 0.43 years (Canada) [50] to 0.74 years (U.S.) [48] and save €7,075 (U.K.) [51] to €19,132 (France) [49] per patient comparing with control over 25 years. When the MiA stage was introduced into the model, early irbesartan remained cost-saving at €2,564 in Hungary [56] to €57,871 in Canada [58] compared with control, being more cost-saving than late irbesartan.

The only study for valsartan [61] also supported the using of ARBs in patients with type 2 diabetes and MiA because of saving QALYs and costs. Over 8 years, valsartan treatment had 0.555 discounted QALYs advantage over amlodipine with savings at €30,424 compared to amlodipine.

## Discussion

To our knowledge, this is the first review that summarizes all information on the cost-effectiveness of both ACEIs and ARBs. Our systematic review confirms earlier results evidencing the cost-saving potentials of ARBs for type 2 diabetic patients with nephropathy compared with conventional therapy excluding a RAAS inhibitor. Also, our review shows that such potentials might even stronger exist in early treatments prior to the stage of nephropathy, for example, in the MiA-stage. In addition, we found similar cost-saving results for ACEIs due to avoidance of ESRD in combination with prolonging life expectancy. Differences in cost effectiveness of ACEI versus ARB could not be solidly established because of differences in model design, time horizon and country setting among all included studies and lack of head-to-head comparisons in economic evaluations. Yet, cost-saving potentials were unequivocally assessed for both drug groups.

### Cost-effectiveness of ACEIs

The number of articles concerning ACEIs was limited compared with the number for ARBs. The reason for not basing studies on single clinical trials may be related to the chronology of ACEIs being available on the market, i.e. the 1980s, before the ARBs. The relevance of CVD in diabetes became only clear in 1990s when the benefits of RAAS inhibitors started also to become clear for diabetic patients. From the six articles included in this review, ACEIs were cost-saving in articles published after 2000s [34-38] and not cost saving (but very cost-effective) in the only one article before 2000s [33]. This may be explained by the patent protection of ACEIs which became generic in the late of 1990s.

Three articles [33,37,38] combined screening for MiA or MaA as the start time point of ACEIs treatment in their analyses. Previous studies of screening for albuminuria with subsequent ACEIs treatment on cardiovascular and renal diseases also support the conclusions on favorable cost-effectiveness and early treatments. Atthobari et al. [71] found that the estimated cost-effectiveness of screening for albuminuria with ACEIs treatment was approximately €16,700/LYG (2006 value) for subjects with a urinary albumin excretion >15 mg/d compared with no screening when adopting the Dutch health care perspective. This was in accordance with the analyses from Boersma C et al. [72] suggesting the potentially favorable cost-effectiveness of population-based screening for MiA compared with other alternatives. Notably, however the latter two articles were for prevention of cardiovascular and renal events in the general population, not particularly for diabetic patients.

### Cost-effectiveness of ARBs

The pharmacoeconomic results of ARBs for renal disease in patients with type 2 diabetes were reviewed previously. Ravera et al. [22] and Boersma et al. [21] reviewed the economic evaluations for ARBs and concluded that evaluations derived from RENAAL, IDNT, IRMA-2 and MARVAL all suggested ARBs to be cost saving compared with conventional therapy in type 2 diabetes patients with nephropathy. Postma & de Zeeuw [26] reviewed the economic benefits of preventing ESRD in patients with type 2 diabetes. They divided the RAAS drug treatment into early and late interventions and concluded that early intervention strategies appear more effective in reducing the risk and the pharmacoeconomic profiles of early intervention clearly outweigh those of late intervention.

From our literature search, there were various economic evaluations on the ARBs losartan, irbesartan and valsartan. There were little differences between studies in each subgroups of ARBs concerning the analysis model, time horizon and measurement of costs and benefits. Although the results varied in different studies and countries, all conclusions supported ARBs as a cost-saving choice.

### Differences in economic evaluations of ACEIs and ARBs

The trials referred to in the studies included in this review had different patient characteristics and treatment strategies. Patients enrolled in ACEIs trials were mainly normotensive, while patients enrolled in ARBs trials were mainly hypertensive. Trails with ACEIs had no equal BP control in placebo groups, whereas trials with ARBs had active BP control in placebo groups. Differences in time horizons used for ACEIs and ARBs present another reason hindering comparison of cost effectiveness between these two drug classes.

Referring to the analytic models, the transition probabilities between two states in the Markov model adopted in these ACEIs studies were from different trials, which may weaken the internal validity of the simulation model used and effectiveness results generated. The analytic models used for ARBs were relatively consistent in their strong alignment to the clinical trials available. Similar methods were adjusted to different country settings. This enhanced similarity in cost-effectiveness results of the same ARB drug in different countries. One might argue that the majority of economic evaluations for losartan were cost analyses with existing trial-based effectiveness as the building block.

### Differences in evaluation results of ACEIs and ARBs

Previous reviews [18-26] of ACEIs and ARBs didn’t summarize the differences between ACEIs and ARBs in the absence of direct comparisons between ARBs and ACE inhibitors in terms of cost-effectiveness. In this review, also no valid comparison between ACEIs and ARBs is possible regarding cost-effectiveness.

In the lifetime treatment for diabetic nephropathy, cost of dialysis when patients develop to ESRD plays an important role in the burden of disease. Comparing to the cost of ESRD, the cost of drugs comprise a relatively low proportion in the total disease expenditure. As ACEIs and ARBs both can delay the deterioration of kidney function to save huge cost due to treatment, results of the economic evaluations included in this review are all pointing into the same direction that these two drug classes are cost-saving or very cost-effective. Furthermore, most ARBs now are available in generic forms and thus cheaper than when these evaluations were performed, which makes ARBs and ACEIs more similar in both effectiveness and cost. Therefore, similar cost-effectiveness result between ACEIs and ARBs can be hypothesized and results in this review strengthen the relevance of the choice made in guidelines [14,18] of recommending ACEIs or ARBs as both presenting cost-effective choices for patients with diabetic nephropathy.

### Limitations

In our review, although the standardized results showed an overview of the cost-effectiveness results of ACEIs and ARBs, to calculate a synthesized economic evaluation result of ACEIs and ARBs using the cost-effectiveness results in different economic evaluations could not validly be done, given all the aforementioned differences. This is mainly due to two limitations. Firstly, the baseline characteristics of the populations varied in the studies included. Secondly, the effectiveness outcomes varied in different studies.

Various selected studies in this review were strongly based on clinical trial settings. Trials are the gold standard for internal validity, but the problem is the lack of external validity [73]. The challenges and the need to include the real-world evidence in economic evaluations has been pointed out by pharmacoeconomic researchers [74]. In the mentioned cost-effectiveness analysis of screening for MiA by Boersma C et al. [72], they used population-based observational data, rather than efficacy data from clinical trials. The obvious problem is these settings is how to adjust for potential confounders and this requires careful consideration. For example, the extent in which data cover the population actually using the drugs, the adverse drug events and the drug use pattern all influence the results of effectiveness analysis. Findings from drug utilization studies relevant to aspects involving (non-)adherence or safety issues should be used in future analyses of drugs’ (cost-)effectiveness. In our efforts to extract some safety information from our current included studies, only two articles [37,38] mention a higher risk of dry cough associated with ACE inhibitors and discuss whether this side effect would influence the cost-effectiveness of ACEIs. This systematic review illustrates the lack of inclusion of observational data in the pharmacoeconomic evaluations so far performed.

## Conclusion

Considering the current evidence, both ACEIs and ARBs are cost-saving compared with conventional therapy excluding a RAAS inhibitors. There is a lack of evidence in direct comparison of these two drug classes in consistent economic evaluations. Because of the limited external validity in using RCT data and the simulation results derived from trial-based analytical models, observational data should be used to confirm these trial-based cost-effectiveness analyses’ results.

## Competing interests

YH works as a PhD student in University Medical Center Groningen (UMCG). Funding was received from University of Groningen. The results of this paper have not been presented or published elsewhere, in whole or in part.

## Authors’ contributions

YH and QZ performed the literature search, data collection and analysis and wrote the draft. YH, FH and MP contributed to the study design, and reviewing the manuscript. All authors contributed to the conception and design, and read and approved the final manuscript.

## Pre-publication history

The pre-publication history for this paper can be accessed here:

http://www.biomedcentral.com/1471-2369/15/15/prepub

## Supplementary Material

Additional file 1 Critical appraisal checklist for economic evaluations. Click here for file
