# Supplementary material for: Economic evaluations of angiotensin-converting enzyme inhibitors and angiotensin II receptor blockers in type 2 diabetic nephropathy: a systematic review
Source: BMC Nephrol. 2014 Jan 15;15:15. doi: 10.1186/1471-2369-15-15 (PMC3913790; doi:10.1186/1471-2369-15-15)
Supplement: Additional file 1 — Critical appraisal checklist for economic evaluations. [file 1471-2369-15-15-S1.doc]

**Additional File 1. Critical Appraisal Checklist for Economic Evaluations**

1. Was a well-defined question posed in an answerable form?

2. Was a comprehensive description of the competing alternatives given (i.e. can you tell who did what to whom, where and how often)?

3. Was there evidence that the programme’s effectiveness had been established?

4. Were all the important and relevant outcomes and costs for each alternative identified?

5. Were outcomes and costs measured accurately in appropriate units (e.g. hours of nursing time, number of physician visits, years-of-life gained) prior to evaluation?

6. Were the outcomes and costs valued credibly?

7. Were outcomes and costs adjusted for different times at which they occurred (discounting)?

8. Was an incremental analysis of the outcomes and costs of alternatives performed?

9. Was a sensitivity analysis performed?

10. Did the presentation and discussion of the results include all, or enough, of the issues that are of concern to purchasers?

11. Were the conclusions of the evaluation justified by the evidence presented?

12. Can the results be applied to the local population?
